# The Synthetic Peptide GA-Hecate and Its Analogs Inhibit Multiple Steps of the Chikungunya Virus Infection Cycle In Vitro

**DOI:** 10.3390/ph16101389

**Published:** 2023-09-30

**Authors:** Gabriela Miranda Ayusso, Paulo Ricardo da Silva Sanches, Tamara Carvalho, Igor Andrade Santos, Daniel Oliveira Silva Martins, Maria Letícia Duarte Lima, Pâmela Jóyce Previdelli da Conceição, Cíntia Bittar, Andres Merits, Eduardo Maffud Cilli, Ana Carolina Gomes Jardim, Paula Rahal, Marilia Freitas Calmon

**Affiliations:** 1 Institute of Biosciences, Letters and Exact Sciences, São Paulo State University, São José do Rio Preto 15054-000, SP, Brazil; gabriela.ayusso@unesp.br (G.M.A.); tamara.carvalho@unesp.br (T.C.); daniel.os.martins@unesp.br (D.O.S.M.); maria-leticia.lima@unesp.br (M.L.D.L.); pamela.joyce@unesp.br (P.J.P.d.C.); cintia.bittar@unesp.br (C.B.); jardim@ufu.br (A.C.G.J.); p.rahal@unesp.br (P.R.); 2 School of Pharmaceutical Sciences, São Paulo State University, Araraquara 14800-903, SP, Brazil; 3 Institute of Biomedical Sciences, Federal University of Uberlândia, Uberlândia 38408-100, MG, Brazil; igorsantos_12@ufu.br; 4 Laboratory of Molecular Immunology, The Rockefeller University, New York, NY 10065, USA; 5 Institute of Technology, University of Tartu, 50090 Tartu, Estonia; andres.merits@ut.ee; 6 Institute of Chemistry, São Paulo State University, Araraquara 14800-060, SP, Brazil; eduardo.cilli@unesp.br

**Keywords:** chikungunya, peptides, analogs, antiviral

## Abstract

Chikungunya virus (CHIKV) belongs to the *Alphavirus* genus and is responsible for significant outbreaks worldwide. Currently, there is no approved antiviral therapy against CHIKV. Bioactive peptides have great potential for new drug development. Here, we evaluated the antiviral activity of the synthetic peptide GA-Hecate and its analogs PSSct1905 and PSSct1910 against CHIKV infection. Initial screening showed that all three peptides inhibited the CHIKV replication cycle in baby hamster kidney fibroblast cells (BHK-21) and human hepatocarcinoma epithelial cells (Huh-7). GA-Hecate and its analog PSSct1905 were the most active, demonstrating suppression of viral infection by more than 91%. The analog PSSct1905 exhibited a protective effect in cells against CHIKV infection. We also observed that the analogs PSSct1905 and PSSct1910 affected CHIKV entry into both cell lines, inhibiting viral attachment and internalization. Finally, all tested compounds presented antiviral activity on the post-entry steps of CHIKV infection in all cells evaluated. In conclusion, this study highlights the potential of the peptide GA-Hecate and its analogs as novel anti-CHIKV compounds targeting different stages of the viral replication cycle, warranting the development of GA-Hecate-based compounds with broad antiviral activity.

## 1. Introduction

Chikungunya virus (CHIKV) is an arbovirus that belongs to the genus *Alphavirus* (family *Togaviridae*). The virions have a single-stranded and positive-sense RNA genome of approximately 11.5 kb in size, which is polyadenylated at the 3′ end and has a 7-methylguanosine cap at the 5′ end. The genome contains two open reading frames (ORFs): (1) a 5′ ORF that encodes a nonstructural polyprotein that is processed into four nonstructural proteins (nsP1, 2, 3, and 4), which are parts of a viral replication complex [1,2], and (2) a 3′ ORF encoding a polyprotein that is processed into five main structural proteins (C, E1, E2, E3, and 6 K) [3,4]. The protein nsP1 demonstrates RNA capping activities and participates in the formation of the viral RNA negative strand; nsP2 triggers blocking of the host transcription process and exhibits RNA helicase, RNA triphosphatase, and proteinase activities; nsP3 recruits RNA-binding proteins that play a role in the formation of CHIKV replication complexes; and nsP4 has RNA-dependent RNA polymerase activity [2,5]. Alphavirus RNA synthesis occurs in replication complexes located in proximity to the plasma membrane [2]. Nonstructural proteins are part of these replication complexes. This complex is responsible for the formation of a full-length negative-strand RNA intermediate, which is used as a template for the production of the positive strand of sub-genomic and genomic RNAs.

The genetic material of CHIKV is surrounded by a capsid (240 copies of C) with icosahedral symmetry, forming the viral nucleocapsid. The CHIKV particle has an envelope derived from the plasma membrane of host cells, to which 80 spikes formed by trimers of glycoproteins E1 and E2 are anchored [6,7]. The viral envelope originates from host cell membranes and contains lipid rafts, sphingolipids, phosphatidylserine, and cholesterol, which provide an amphipathic character and negative charge. CHIKV entry into cells is mediated by the viral glycoproteins E2 and E1 through several attachment factors—namely, heparan sulfate and phosphatidylserine receptors (TIM1 [8], TIM4, and AXL [9])—and receptors such as Mxra8 [10]. The viral glycoprotein E2 is responsible for interacting with cell receptors, including Mxra8, the main receptor for several arthritogenic alphaviruses, including CHIKV [10,11]. The CHIKV cell receptor is ubiquitously expressed in several species and cell types [12,13]. Importantly, the binding of CHIKV virions is facilitated by several other cellular components. Glycosaminoglycans (GAGs), prohibitin (PHB), and phosphatidylserine (PtdSer)-mediated virus entry-enhancing receptors (PVEERs) are cited in the literature as factors of CHIKV attachment in mammalian cells [14,15,16]. CHIKV enters cells by clathrin-mediated endocytosis. Subsequently, the fusion of the viral and endosomal membranes occurs in a low-pH-dependent manner [17].

CHIKV was first reported during an outbreak that occurred in Tanzania in 1953. Currently, this arbovirus is fully adapted to the urban transmission cycle, raising concern in many tropical and temperate regions [18]. Although CHIKV continues to cause significant outbreaks around the world to date, there are no specific antiviral treatments or licensed vaccines against this viral infection [19]. Several antiviral strategies against CHIKV are being evaluated, mainly in vitro, that can affect critical steps of the viral replication cycle, such as binding and entry, viral genome replication, functionality of viral proteins, virion formation, and infectivity [20,21,22,23,24].

Antiviral peptides that interact with virus particles or target other critical steps of viral infection could potentially be used as treatment or prophylaxis for virus infection [25,26]. Most antiviral peptides are short (10 to 50 amino acid residues), have a positive charge, and have both hydrophilic and hydrophobic characteristics [27,28]. These peptides usually act at different steps of the virus replication cycle [26]. For example, the peptide (p-BthTX-I)_2_K derived from *Bothrops jararacussu* snake impaired CHIKV entry into baby hamster kidney fibroblast cells by inhibiting the attachment and internalization steps. Additionally, the same peptide also inhibited Zika virus (ZIKV) infection by interfering with the post-entry stages of the viral replication cycle, reducing the viral protein NS3, which is essential for ZIKV replication [29]. LL-37, a peptide of the Cathelicidin family, impacts the early processes of Venezuelan equine encephalitis virus (VEEV) infection [30]. Two synthetic peptides, A2 and A3, inhibited replication of VEEV and eastern equine encephalitis virus (EEEV) in vitro, and both peptides, inhibited VEEV attachment to cells [31]. Surfactin, a cyclic lipopeptide, significantly reduced the infectivity of CHIKV, Mayaro virus, and Una virus in vitro [32]. Antiviral peptides have other mechanisms of action, such as disruption of the virus envelope, blocking release in host cells, and total or partial inhibition of viral particle assembly [21,25,28].

Different studies have demonstrated that peptide conjugation is a relevant strategy for the development of new substances and increasing their biological activity [25,33,34]. The peptide Hecate (FALALKALKKALKKLKKALKKAL-CONH_2_) is a synthetic peptide that has a net positive charge and amphipathic characteristics, exhibiting many biological activities, with emphasis on antiviral and antitumor activity [35,36]. Gallic acid (GA) (C_7_H_6_O_5_) is a phenolic compound found in abundance in plants that exhibits antiviral, antibacterial, and antitumor activities [37,38]. Our group has evaluated the anti-hepatitis C virus (HCV) activity of five analogs of Hecate in human hepatocellular carcinoma-derived Huh-7.5 cells. The peptide conjugated to GA (GA-Hecate) represented the most active compound, inhibiting the main stages of the HCV replication cycle. Thus, the antiviral properties of GA-Hecate encourage its use as a prototype for the development of new antivirals [35].

This study aimed to evaluate the potential of GA-Hecate and its analogs PSSct1905 (GA-FALALKALKKALKKL-COOH) and PSSct1910 (4-(dimethylamino)-benzoic acid-FALALKALKKALKKLKKALKKAL-CONH_2_) as antivirals against CHIKV. The study was performed by testing the effects of these peptides on different steps of the CHIKV infectious cycle.

## 2. Results and Discussion

### 2.1. Cytotoxicity of the Peptides

**Figure 1:**
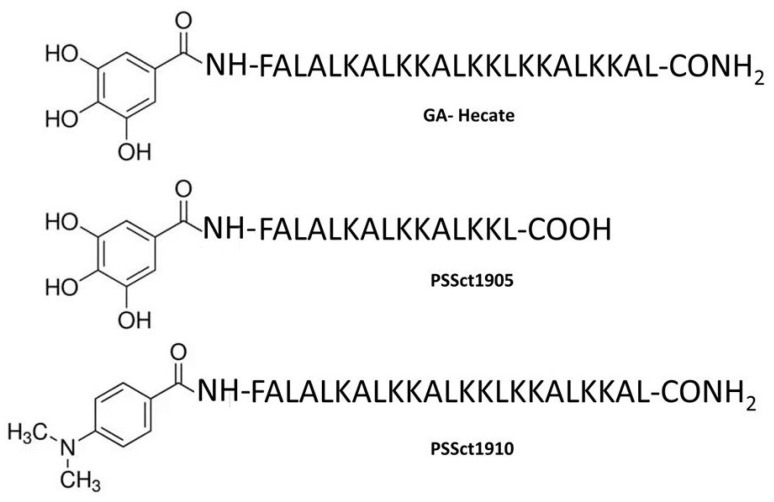
Structures of the peptide GA-Hecate and its analogs PSSct1905 and PSSct1910.

The structures of the peptide GA-Hecate and its analogs PSSct1905 and PSSct1910 are presented in Figure 1. The physicochemical properties and biological activities of these peptides are summarized in Table 1. The cytotoxicity of the peptides GA-Hecate, PSSct1905, and PSSct1910 was evaluated in baby hamster kidney fibroblast cells (BHK-21) and human hepatocarcinoma epithelial cells (Huh-7). We defined the maximum nontoxic concentration (MNTC), expressed in µM, as the highest peptide concentration that kept at least 80% of BHK-21 and Huh-7 cells viable within 24 h.

All peptides had higher 50% cytotoxic concentration (CC_50_) values for Huh-7 cells than for BHK-21 cells. The MNTC of GA-Hecate and its analog PSSct1905 was 12.5 µM or higher in these cell types (Figure S1A,B,D,E). Compared to GA-Hecate, the peptide PSSct1905 lacks the last eight amino acids in the C-terminus, a modification made on the basis of a previous observation that these amino acids were susceptible to degradation by blood proteases, decreasing the stability of the compound. Based on our analysis, this modification resulted in a modest (approximately 2-fold) increase in the CC_50_ value of the compound; the difference was more prominent in BHK-21 cells. The analog PSSct1910 was considerably more cytotoxic, with CC_50_ values of 5.4 µM and 7.7 µM for BHK-21 and Huh-7 cells, respectively. For both cell lines, the MNTC was 1.6 µM (Figure S1C,F). These data indicate that the only structural change that promoted higher cytotoxicity was the replacement of GA by the (4-(dimethylamino)-benzoic acid) subunit at the N-terminal end of the peptide GA-Hecate. Additional studies of the structure-activity relationship should be performed to better understand the role of these modifications in the cytotoxic effects of these compounds.

### 2.2. The Peptides Strongly Impaired the Replication Cycle of CHIKV

**Figure 2:**
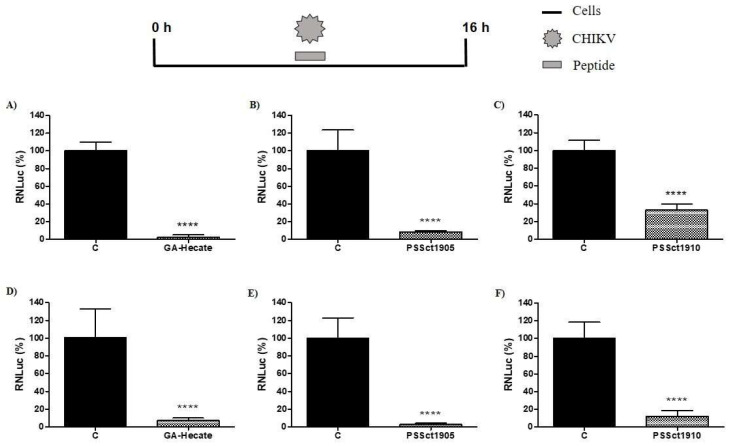
Inhibitory effect of the peptides on the CHIKV replication cycle in BHK-21 and Huh-7 cells. The percentages were expressed in relation to protein NanoLuciferase (NLuc) activity in cells treated with the control (C, sterile water). Luminescence signals were measured in three independent experiments, each performed in quadruplicate, and data are presented as the mean ± standard deviation (SD). (**A**) GA-Hecate at the MNTC (12.5 µM) in BHK-21 cells. (**B**) PSSct1905 at the MNTC (12.5 µM) in BHK-21 cells. (**C**) PSSct1910 at the MNTC (1.6 µM) in BHK-21 cells. (**D**) GA-Hecate at the MNTC (12.5 µM) in Huh-7 cells. (**E**) PSSct1905 at the MNTC (12.5 µM) in Huh-7 cells. (**F**) PSSct1910 at the MNTC (1.6 µM) in Huh-7 cells. ****: *p* ≤ 0.0001.

The peptides GA-Hecate, PSSct1905, and PSSct1910 were tested at the MNTC in BHK-21 and Huh-7 cells infected with CHIKV-expressing *nanoluciferase* reporter (CHIKV-NLuc) [multiplicity of infection (MOI) 0.1] for 16 h. All three peptides exhibited significant antiviral effects against the replication cycle of CHIKV. GA-Hecate was the most active, causing inhibition of viral replication of 97.7% (*p* ≤ 0.0001) in BHK-21 cells and 93.6% (*p* ≤ 0.0001) in Huh-7 cells when compared to the control (Figure 2A,D). Among the analogs, PSSct1905 also showed a high level of inhibition of the CHIKV replicative cycle; the effect was slightly more prominent in Huh-7 cells (97.3% inhibition; *p* ≤ 0.0001) than in BHK-21 cells (91.7% inhibition; *p* ≤ 0.0001) (Figure 2B,E). Similar activities imply that the removal of KKALKKAL amino acids from the C-terminal end of GA-Hecate has minimal effect on the antiviral activity of the compound. On the other hand, the replacement of the GA by the group with (4-(dimethylamino)-benzoic acid at the N-terminal end of the peptide GA-Hecate negatively impacted its inhibitory potential; peptide PSSct1910, harboring such a modification, resulted in 67.2% inhibition (*p* ≤ 0.0001) of CHIKV replication in BHK-21 cells and 87.8% inhibition (*p* ≤ 0.0001) in Huh-7 cells (Figure 2C,F). However, it should be noted that, due to its increased cytotoxicity, this compound was also used at a much lower concentration than the other two inhibitors. Indeed, PSSct1910 treatment resulted in the lowest 50% effective concentration (EC_50_) values for CHIKV inhibition, with a higher inhibitory effect (approximately 5-fold) in Huh-7 cells (EC_50_ = 0.2 µM) than in BHK-21 cells (EC_50_ = 1.1 µM). Thus, it may still be an efficient inhibitor. All peptides had a higher selectivity index (SI) in Huh-7 cells than in BHK-21 cells. GA-Hecate was the most selective in Huh-7 cells, resulting in an SI value of 49.1.

The peptide Hecate can bind with lipids, disrupting lipid bilayers [39]. Hecate’s ability to bind with lipids can trigger changes in the fluidity of the virus envelope, cause disturbances in viral envelope formation, and interfere with viral fusion and/or egress in host cells [40]. Indeed, Hecate showed antiviral activity against an unrelated enveloped virus, herpes simplex virus (HSV-1), infection in African green monkey kidney cells (Vero) by inhibiting cell fusion [41]. GA also inhibited the replication cycle of HSV in Vero cells [42]. It has also been demonstrated that this compound significantly reduced the infection of o’nyong-nyong virus (ONNV), an alphavirus closely related to CHIKV, in human skin fibroblast (HSF) cells [43]. GA exhibits antiviral activity through different mechanisms of action, such as virucidal and protective effects and interference with virus entry and replication in host cells [37,44,45]. GA-Hecate inhibited the efficiency of multiple steps in the HCV replication cycle in Huh-7.5 cells [35]. These data indicate that GA-Hecate and its analogs may impair the CHIKV replication cycle due to interference with more than one step of the CHIKV infection cycle. For this reason, we decided to evaluate which phase of the CHIKV replication cycle these peptides could inhibit.

### 2.3. Antiviral Effect of Pretreatment with the Peptides against CHIKV

**Figure 3:**
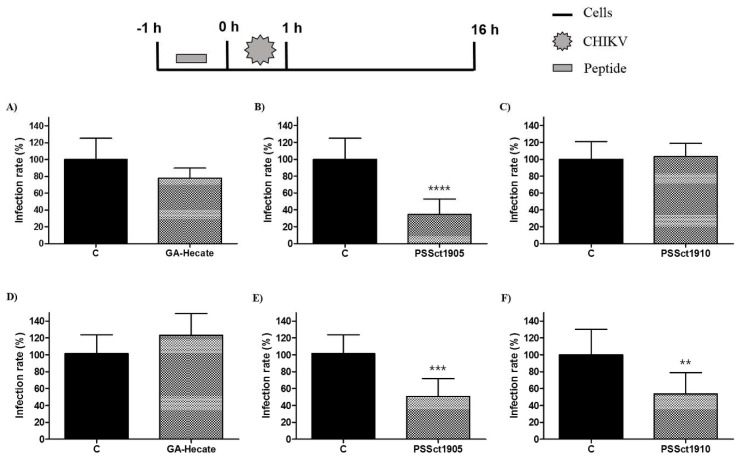
Protective effect of the peptides against CHIKV infection. Data were analyzed as described in Figure 2. Luminescence signals were measured from three independent experiments, each performed in quadruplicate, and data are presented as the mean ± SD. (**A**) GA-Hecate in BHK-21 cells. (**B**) PSSct1905 in BHK-21 cells. (**C**) PSSct1910 in BHK-21 cells. (**D**) GA-Hecate in Huh-7 cells. (**E**) PSSct1905 in Huh-7 cells. (**F**) PSSct1910 in Huh-7 cells. C: sterile water. **: *p* ≤ 0.01; ***: *p* ≤ 0.001; ****: *p* ≤ 0.0001.

To evaluate the protective effect of pretreatment of cells against CHIKV infection, BHK-21 and Huh-7 cells were treated with the peptides at the MNTC for 1 h, washed with PBS (phosphate-buffered saline), and infected with CHIKV-NLuc (MOI 0.1). Pretreatment of cells with GA-Hecate did not result in detectable protection against CHIKV infection (Figure 3A,D), while its analog PSSct1905 was able to significantly protect BHK-21 and Huh-7 cells, reducing CHIKV infection by 65.4% (*p* ≤ 0.0001) and 50.6% (*p* ≤ 0.001), respectively (Figure 3B,E). Pretreatment with PSSct1910 had a protective effect against CHIKV in Huh-7 cells (inhibition by 46.1%; *p* ≤ 0.01) but not in BHK-21 cells (Figure 3C,F).

Taken together, pretreatment of cells with peptides revealed that protection of cells by prophylactic treatment is not the main mechanism of action of these antiviral peptides. Nevertheless, some modest antiviral activity was observed. CHIKV infection starts with the binding of virus particles to the cellular surface, triggering attachment, and internalization [46,47]. All tested peptides also have positive charges, and, probably, in the pretreatment, the positive charges facilitate their binding to the cell. This binding may inhibit the interaction between the E2 glycoprotein and molecules on the cell surface and, consequently, the CHIKV attachment and internalization in the host cell. However, the positive charge of peptides alone could not explain all observed effects, as GA-Hecate is also positively charged yet offers no protection. It is plausible that the size difference between this peptide and the analog PSSct1905 may be responsible for different interactions with CHIKV attachment factors/receptors and these differences alter the ability of the compound to protect cells against infection.

### 2.4. Virucidal Effect of the Peptides on CHIKV Virions

**Figure 4:**
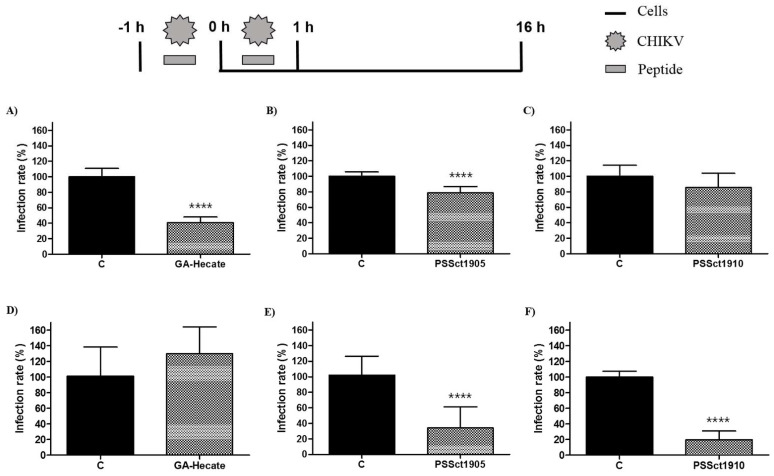
Effect of the peptides-treated CHIKV virions on BHK-21 and Huh-7 cells. Data were analyzed as described in Figure 2. Luminescence signals were measured from three independent experiments, each performed in quadruplicate, and data are presented as the mean ± SD. (**A**) GA-Hecate in BHK-21 cells. (**B**) PSSct1905 in BHK-21 cells. (**C**) PSSct1910 in BHK-21 cells. (**D**) GA-Hecate in Huh-7 cells. (**E**) PSSct1905 in Huh-7 cells. (**F**) PSSct1910 in Huh-7 cells. C: sterile water. ****: *p* ≤ 0.0001.

To investigate the ability of peptides GA-Hecate, PSSct1905, and PSSct1910 to inactivate extracellular CHIKV virions, the virus particles were treated with the peptides at the MNTC for 1 h and used for infection of BHK-21 and Huh-7 cells. In this assay, the peptides GA-Hecate and PSSct1905 reduced infection in BHK-21 cells, showing inhibitory effects of 59.4% (*p* ≤ 0.0001) and 21.4% (*p* ≤ 0.0001), respectively (Figure 4A,B). In Huh-7 cells, GA-Hecate did not inhibit viral infection (Figure 4D), while treatment with PSSct1905 resulted in 68% (*p* ≤ 0.0001) inhibition (Figure 4E). The peptide PSSct1910, on the other hand, only presented virucidal activity when treated virions were used to infect Huh-7 cells (80.2% inhibition; *p* ≤ 0.0001) (Figure 4F).

The peptides used are positively charged and amphipathic; these properties may play important roles in their activity against enveloped particles, such as the virions of alphaviruses. Amphipathic cationic peptides can interact with this virus envelope and produce a direct virucidal effect, resulting in interference with virus binding and/or fusion [48]. Similarly, a study by Ahmed and collaborators observed that a cationic peptide can interfere with virus binding by causing the aggregation of extracellular virions [30]. Therefore, it is plausible that the peptides used caused the inactivation of CHIKV virions by disrupting the viral envelope. Indeed, it was observed by Batista and coworkers that the peptide GA-Hecate was capable of generating disruption of the HCV viral mimetic envelope [35]. Interestingly, however, in this study, the impact of virion treatment clearly depended on the cell line that was infected with peptide-treated particles; for GA-Hecate, no effect of peptide treatment was observed upon infection of Huh-7 cells, while treatment with PSSct1905 or PSSct1910 had no or minimal impact on the ability of CHIKV virions to infect BHK-21 cells. These data argue against the possibility of general destruction (or aggregation) of virions and are more consistent with milder peptide-induced damage. If so, the difference in the expression of receptors on the BHK-21 and Huh-7 cell surfaces and their affinity toward CHIKV could explain this difference. Furthermore, the experimental setup included 1 h of incubation of cells with inoculum containing both virus particles and peptides. Thus, it is possible that in the presence of peptides, the binding of virions to cells or their entry was also impaired in a cell-type-dependent manner. Therefore, the impact of peptides on these steps of the virus infection cycle was subsequently analyzed.

### 2.5. Antiviral Activity of the Peptides on the CHIKV Entry Steps

**Figure 5:**
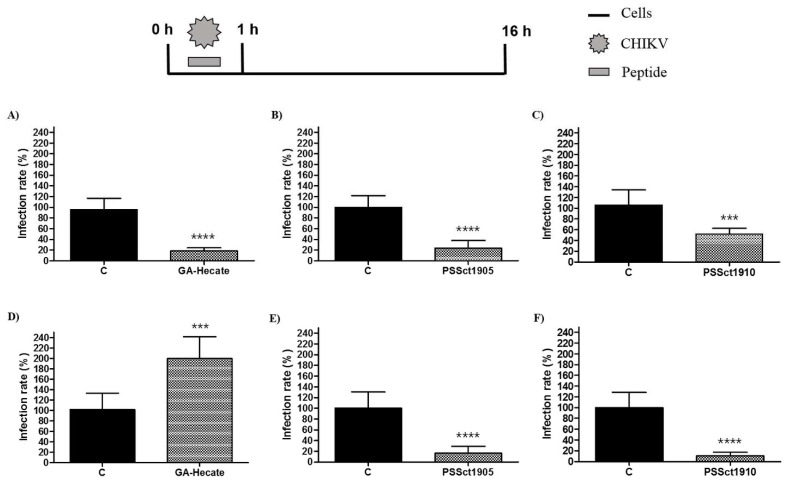
Effect of the peptides on CHIKV entry. Data were analyzed as described in Figure 2. Luminescence signals were measured from three independent experiments, each performed in quadruplicate, and data are presented as the mean ± SD. (**A**) GA-Hecate in BHK-21 cells. (**B**) PSSct1905 in BHK-21 cells. (**C**) PSSct1910 in BHK-21 cells. (**D**) GA-Hecate in Huh-7 cells. (**E**) PSSct1905 in Huh-7 cells. (**F**) PSSct1910 in Huh-7 cells. C: sterile water. ***: *p* ≤ 0.001; ****: *p* ≤ 0.0001.

To evaluate the action of the peptides in the early steps of the CHIKV replication cycle, BHK-21 and Huh-7 cells were infected with CHIKv-NLuc at an MOI of 0.1 in the presence of the MNTC of GA-Hecate, PSSct1905, or PSSct1910 for 1 h, washed with PBS, and incubated with peptide-free culture medium. In this experiment, all peptides inhibited CHIKV entry into BHK-21 cells. The effect was strongest for GA-Hecate and PSSct1905, for which 77.2% (*p* ≤ 0.0001) and 76.4% (*p* ≤ 0.0001) inhibition were observed, respectively (Figure 5A,B). PSSct1910 was somewhat less potent, with an inhibition rate of 54.1% (*p* ≤ 0.001) compared to the control (Figure 5C). Curiously, this trend is the same as that observed in the previous experiment (Figure 4A–C); the different inhibition rates observed in these experiments can be attributed to the 50-fold higher MOI used in the previous experiment, which makes the assay less sensitive. In Huh-7 cells, GA-Hecate failed to inhibit the early steps of CHIKV infection; instead, significant activation was observed (Figure 5D). At the same time, PSSct1905 and PSSct1910 reduced the efficiency of the entry stages of CHIKV infection and resulted in 84% (*p* ≤ 0.0001) and 89.5% (*p* ≤ 0.0001) inhibition, respectively (Figure 5D–F). Again, the trend is similar to that observed in the previous experiment (Figure 4D–F). Combined with data obtained for BHK-21 cells, these results strongly indicate that antiviral activities observed in the previous experiment were mostly, if not exclusively, due to inhibition of early stages of CHIKV infection. Hence, it is unclear whether the peptides have virucidal effects. In order to prove the existence or absence of virucidal properties, additional experiments would be needed.

### 2.6. Antiviral Effect of the Peptides on CHIKV Attachment

To reveal which processes involved in the entry of CHIKV infection were affected by the peptides, we first investigated their activity on CHIKV attachment. In this experiment, BHK-21 and Huh-7 cells were incubated with peptides at the MNTC and CHIKV-NLuc in amounts corresponding to infection at an MOI of 0.1 at 4 °C. At this temperature, virions interact with cell membrane receptors, but the viral particle is not able to enter the cell [49]. Subsequently, the cells were washed with PBS and incubated with a culture medium to continue the viral entry process. As GA-Hecate was unable to inhibit CHIKV entry into Huh-7 cells (Figure 5D), this combination was excluded from this analysis.

**Figure 6:**
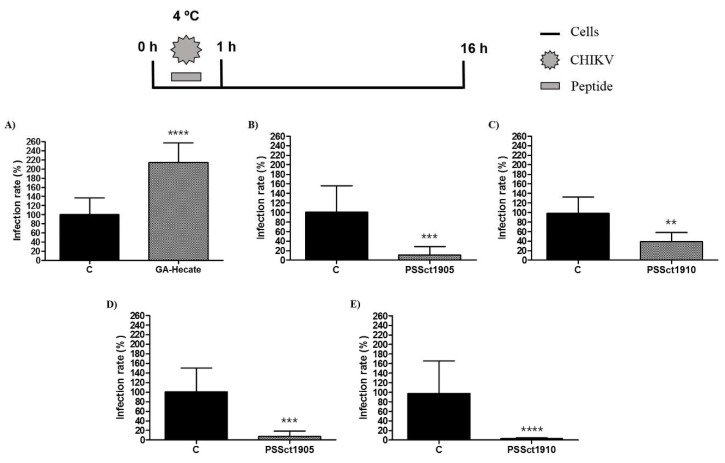
Effect of the peptides on CHIKV attachment. Data were analyzed as described in Figure 2. Luminescence signals were measured from three independent experiments, each performed in quadruplicate, and data are presented as the mean ± SD. (**A**) GA-Hecate in BHK-21 cells. (**B**) PSSct1905 in BHK-21 cells. (**C**) PSSct1910 in BHK-21 cells. (**D**) PSSct1905 in Huh-7 cells. (**E**) PSSct1910 in Huh-7 cells. C: sterile water. **: *p* ≤ 0.01; ***: *p* ≤ 0.001; ****: *p* ≤ 0.0001.

Despite demonstrating an antiviral effect on the entry of CHIKV into BHK-21 cells, the peptide GA-Hecate did not inhibit the viral attachment step (Figure 6A). These data indicate that the peptide GA-Hecate probably inhibits viral entry by interfering with the internalization of CHIKV in BHK-21 cells. In contrast, PSSct1905 and PSSct1910 inhibited the attachment of CHIKV virions to BHK-21 and Huh-7 cells. In BHK-21 cells, treatment with PSSct1910 exhibited less prominent antiviral activity (59.9% inhibition; *p* ≤ 0.01) than PSSct1905, which resulted in 89.5% inhibition (*p* ≤ 0.001) (Figure 6B,C). In Huh-7 cells, treatment with PSSct1905 or PSSct1910 resulted in very high levels of inhibition with minimal differences between these compounds: 93.5% (*p* ≤ 0.001) and 95.3% (*p* ≤ 0.0001) inhibition, respectively (Figure 6D,E). Interestingly, these effects were more prominent than those obtained for the inhibition of the complete entry stage (Figure 5B,C,E,F); the difference is probably due to different experimental conditions, such as the low temperature used in the assay that analyzed the effect of peptides on the attachment step.

Cationic peptides can impair virus attachment to host cells by blocking cell receptors and/or by binding to virions [50]. As cited earlier, the peptides in this study have a positive charge similar to the E2 glycoprotein of the CHIKV. This may lead to competition for binding to cellular receptors, resulting in reduced attachment of virions to the cell.

### 2.7. Antiviral Action of the Peptides on CHIKV Internalization

In addition to attachment, the peptides GA-Hecate, PSSct1905, and PSSct1910 were tested for their ability to inhibit CHIKV internalization in BHK-21 and Huh-7 cells. For this analysis, the cells were first incubated with viral particles at 4 °C to allow viral attachment to occur; after this, the inoculum was replaced with media containing the peptides at the MNTC, and cells were placed at 37 °C to allow the internalization of the CHIKV particles. After 1 h, the cells were again washed with PBS and incubated with culture medium to continue the replicative cycle of the virus. As in the previous experiment, the combination of GA-Hecate and Huh-7 cells was excluded from this analysis.

**Figure 7:**
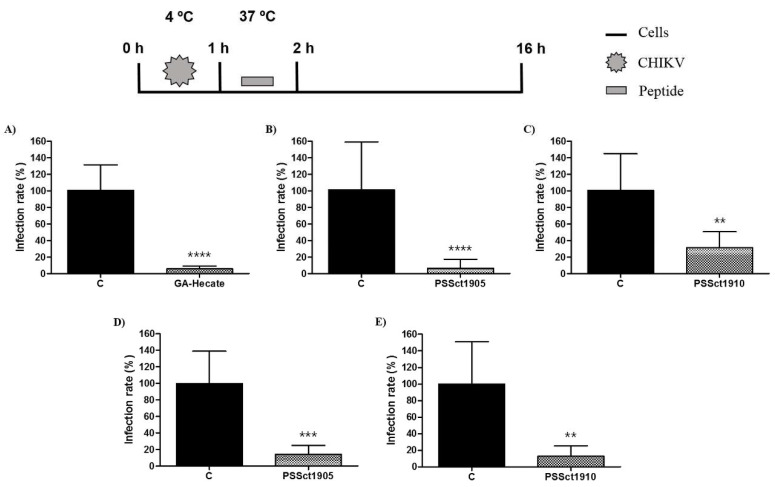
Effect of the peptides on CHIKV internalization. Data were analyzed as described in Figure 2. Luminescence signals were measured from three independent experiments, each performed in quadruplicate, and data are presented as the mean ± SD. (**A**) GA-Hecate in BHK-21 cells. (**B**) PSSct1905 in BHK-21 cells. (**C**) PSSct1910 in BHK-21 cells. (**D**) PSSct1905 in Huh-7 cells. (**E**) PSSct1910 in Huh-7 cells. C: sterile water. **: *p* ≤ 0.01; ***: *p* ≤ 0.001; ****: *p* ≤ 0.0001.

The peptide GA-Hecate, which was unable to inhibit the attachment of CHIKV to BHK-21 cells (Figure 6A), strongly inhibited the internalization of CHIKV particles into BHK-21 cells. The observed inhibition was approximately 95.1% (*p* ≤ 0.0001) (Figure 7A). This inhibition is apparently prominent enough to overcome the activation of CHIKV attachment, resulting in inhibition, albeit more modest, of the CHIKV entry step in general (Figure 5A). The viral internalization stage was also inhibited by PSSct1905 and PSSct1910 treatment, resulting in the inhibition of 94.5% (*p* ≤ 0.0001) and 69.1% (*p* ≤ 0.01), respectively, in BHK-21 cells (Figure 7B,C). High levels of inhibition were also observed in Huh-7 cells, in which treatment with PSSct1905 and PSSct1910 resulted in 85.8% (*p* ≤ 0.001) and 87.1% (*p* ≤ 0.01) inhibition, respectively (Figure 7D,E). Thus, regardless of the cell type, these analogs inhibited CHIKV entry by interfering with both virus attachment and internalization. Whether these two mechanisms of action can result in additive (or cumulative) effects remains unclear, as the use of different experimental conditions (most notable use of different temperatures) in assays that evaluate entry in general and attachment/internalization stages specifically does not allow direct comparison of data originating from corresponding experiments.

Amphipathic and positively charged peptides have been described to inhibit the intracellular trafficking of many pH-dependent enveloped viruses [51]. It is plausible that the peptides used in this study enter the cells together with the virions using endocytic vesicles by binding to cell (and/or virion) surfaces. In endocytic vesicles, they can suppress endosomal acidification, delaying or preventing the activation of pH-triggered viral fusogenic machinery, which is crucial for viral genome release into the cytoplasm. Additionally, it is possible that peptides hinder acidification via their electrostatic contributions to the endosomal lumen, resulting in resistance to the influx of hydrogen ions (driven by H^+^ ATPase pumps). Alternatively, peptides may partially act as a buffering agent by sequestering incoming luminal protons. Finally, the peptides might regulate endosomal acidification by directly interacting with the host machinery, which drives this process [52].

### 2.8. Antiviral Activity of the Peptides on the Post-Entry Steps of CHIKV Infection

**Figure 8:**
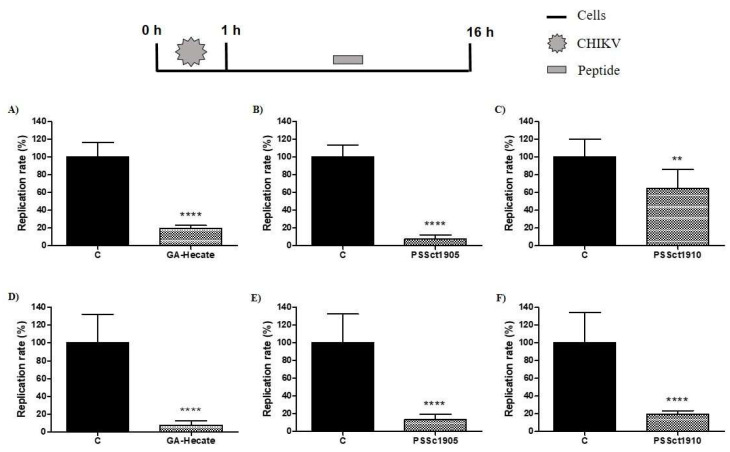
Effect of the peptides on the post-entry steps of CHIKV infection. Data were analyzed as described in Figure 2. Luminescence signals were measured from three independent experiments, each performed in quadruplicate, and data are presented as the mean ± SD. (**A**) GA-Hecate in BHK-21 cells. (**B**) PSSct1905 in BHK-21 cells. (**C**) PSSct1910 in BHK-21 cells. (**D**) GA-Hecate in Huh-7 cells. (**E**) PSSct1905 in Huh-7 cells. (**F**) PSSct1910 in Huh-7 cells. C: sterile water. **: *p* ≤ 0.01; ****: *p* ≤ 0.0001.

To evaluate the antiviral activity of the peptides in the post-entry stages of the CHIKV replicative cycle, BHK-21 and Huh-7 cells were infected with CHIKV-NLuc at an MOI of 0.1. After 1 h of incubation, the cells were washed, and media containing peptides at the MNTC was added. In this assay, GA-Hecate inhibited CHIKV infection by 80.7% (*p* ≤ 0.0001) and 92.7% (*p* ≤ 0.0001) in BHK-21 and Huh-7 cells, respectively (Figure 8A,D). The peptide PSSct1905 caused 92.2% (*p* ≤ 0.0001) and 86.8% (*p* ≤ 0.0001) reductions in viral replication in BHK-21 and HuH-7 cells, respectively (Figure 8B,E). The lowest activities were observed for the peptide PSSct1910, which reduced viral replication by approximately 35.2% (*p* ≤ 0.01) in BHK-21 cells and 80.3% (*p* ≤ 0.0001) in Huh-7 cells (Figure 8C,F). We suggest that the peptides used in this study may interfere with the synthesis of the CHIKV nonstructural proteins or inhibit their activity. The analysis of potential targets was outside of the scope of the current study.

## 3. Materials and Methods

### 3.1. Peptides

GA-Hecate and its analogs PSSct1905 and PSSct1910 were prepared on a TRIBUTE-UV automatic synthesizer (Protein Technologies, Tucson, AZ, USA) via solid phase peptide synthesis (SPPS) using the standard Fmoc protocol (9-fluorenylmethyloxycarbonyl) on a Rink-MBHA resin for the peptides GA-Hecate and PSSct1910 and Fmoc-Leu-Wang resin for PSSct1905 [36]. The identities of the peptides were confirmed by electrospray ionization mass spectrometry, and their purities were higher than 95%. To perform the biological experiments, the peptides were dissolved in sterile water and stored at –150 ºC.

### 3.2. Cells

BHK-21 cells (ATCC CCL-10) and Huh-7 cells were cultured in Dulbecco’s modified Eagle’s medium (DMEM, Cultilab, Campinas, SP, Brazil) supplemented with 10% fetal bovine serum (FBS, Gibco—Thermo Fisher Scientific, Waltham, MA, USA) and 1% penicillin (10,000 IU/mL)/streptomycin (10 mg/mL) (P/S, Cultilab, Campinas, SP, Brazil). The cells were maintained in a humidified incubator at 37 °C in 5% CO_2_.

### 3.3. Virus

The CHIKV-NLuc-expressing *nanoluciferase* reporter used for the antiviral assays is based on the CHIKV isolate LR2006OPY1 (East/Central/South African genotype). In the infectious plasmid, the cDNA of CHIKV-NLuc was placed under the control of the human cytomegalovirus (CMV) promoter [53]. To rescue CHIKV-NLuc, 1 × 10^5^ BHK-21 cells seeded in 24-well culture plates (TPP, Trasadingen, Switzerland) were transfected with 1 μg of CHIKV-NLuc plasmid DNA using Lipofectamine 2000 (Thermo Fisher Scientific, Waltham, MA, USA) and OPTI-MEM (reduced serum medium) (Gibco—Thermo Fisher Scientific, Waltham, MA, USA), as previously described [54]. The supernatant was collected at 72 h post-transfection (h.p.t.) and stored at –80 °C.

The viral titers were determined by plaque assay, following the previously described protocol [54]. BHK-21 cells (1 × 10^5^) were seeded in 24-well plates (TPP, Trasadingen, Switzerland). Twenty-four hours later, the cells were infected with tenfold serial dilutions of CHIKV-NLuc and incubated for 1 h at 37 °C. After this, the inoculums were removed and replaced with fresh medium supplemented with 1% P/S (Cultilab, Campinas, SP, Brazil), 1% FBS (Gibco—Thermo Fisher Scientific, Waltham, MA, USA), and 2% carboxymethylcellulose (CMC, Sigma-Aldrich, St. Louis, MO, USA). Infected cells were incubated for 48 h in a humidified 5% CO_2_ incubator at 37 °C, followed by fixation with 10% formaldehyde (Merck, Darmstadt, Germany) and staining with 1% violet crystal (Merck, Darmstadt, Germany). The viral foci were counted to determine the viral titer, which was presented in plaque-forming units per milliliter (PFU/mL).

### 3.4. Evaluation of the Cytotoxicity Profile of the Peptides

Cytotoxicity in BHK-21 and Huh-7 cells was evaluated using the 3-(4,5-dimethylthiazol-2-yl)-2,5-diphenyltetrazolium bromide (MTT) assay as previously described [35]. The cells (5 × 10^3^ per well) were seeded in 96-well culture plates (TPP, Trasadingen, Switzerland). After 24 h, the cells were incubated with the peptides at concentrations of 1.6, 3.1, 6.3, 12.5, 25, 50, and 100 µM for 24 h. Then, the medium containing the peptides was removed, and 100 µL of MTT (Sigma-Aldrich, St. Louis, MO, USA) diluted in DMEM (Cultilab, Campinas, SP, Brazil) (1 mg/mL) was added to each well of the plate (1 mg/mL). After 30 min of incubation at 37 °C, the medium containing MTT (Sigma-Aldrich, St. Louis, MO, USA) was removed, and 100 µL of dimethylsulfoxide (DMSO, Synth, Diadema, SP, Brazil) was added to the cells. The plate was agitated at 200 rpm. After 5 min, the absorbance was measured at a wavelength of 572 nm on a plate reader (FLUOstar Omega/BMG LABTECH, Ortenberg, Germany). The CC_50_ values were calculated using GraphPad Prism 5.0 software (GraphPad Software, San Diego, CA, USA).

### 3.5. Analysis of the Activity of the CHIKV-NLuc-Encoded Reporter

Measurement of the activity of the virus-encoded NanoLuciferase (NLuc) reporter was used to evaluate the antiviral activity of the peptides against CHIKV-NLuc at the end of every assay with the peptides. Cells were infected with CHIKV-NLuc, and the supernatant was aspirated 16 h post-infection (h.p.i.), and each well of the plate was washed with PBS solution. Then, 30 µL of Renilla Luciferase Assay Lysis Buffer (Promega, Madison, WI, USA) was added to the cells. After 30 min, 50 μL of substrate for Renilla Luciferase (Renilla Luciferase assay reagent, Promega, Madison, WI, USA) was automatically injected in the wells into the plate reader (FLUOstar Omega/BMG LABTECH, Ortenberg, Germany), and the light intensity reading was performed. The values obtained are shown as the percentage of protein NLuc activity in the control (samples treated with sterile water).

### 3.6. Evaluation of the Activity of Peptides against the CHIKV Replication Cycle

The effects of the peptides on the CHIKV infectious cycle were analyzed as previously described [55]. BHK-21 and Huh-7 cells (1 × 10^4^ per well) were seeded in 96-well white culture plates (Greiner Bio-One, Americana, SP, Brazil) for 24 h. Then, the virus at an MOI of 0.1 and the peptides at the MNTC were simultaneously added to the cells. The plate was incubated at 37 °C for 16 h, and then antiviral activity against CHIKV was evaluated through NLuc activity. The peptides that exhibited antiviral activity at the MNTC were tested at concentrations of 1.6, 3.1, 6.3, 12.5, 25, 50, and 100 µM to determine the EC_50_ values, which were calculated using GraphPad Prism 5.0 software (GraphPad Software, San Diego, CA, USA). The SI was calculated using the ratio CC_50_/EC_50_.

### 3.7. Analysis of the Protective Effect of the Peptides against CHIKV Infection in the Pretreatment Assay

The protective effect of the peptides against CHIKV infection was evaluated as previously described [55]. BHK-21 and Huh-7 cells (1 × 10^4^ per well) were seeded in 96-well white culture plates (Greiner Bio-One, Americana, SP, Brazil). After 24 h, the cells were treated with peptides at the MNTC for 1 h at 37 °C. Then, the supernatant was removed, each well of the plate was washed twice with PBS, and the virus at an MOI of 0.1 was added to the cells and incubated for 1 h at 37 °C. After that, cells were washed twice with PBS again, and DMEM (Cultilab, Campinas, SP, BR) supplemented with 2% FBS (Gibco—Thermo Fisher Scientific, Waltham, MA, USA) was added to the cells. The plate was incubated at 37 °C, and the protective effect against CHIKV infection was evaluated at 16 h.p.i. by measurement of NLuc activity.

### 3.8. Evaluation of the Effect of the Peptides on the Extracellular CHIKV Particles

The virucidal activity of the peptides against CHIKV virions was investigated as previously described [56]. BHK-21 and Huh-7 cells (1 × 10^4^ per well) were seeded in 96-well white culture plates (Greiner Bio-One, Americana, SP, Brazil). A total of 5 × 10^4^ PFU of virus was incubated with the MNTC of peptides at 37 °C for 1 h, after which the viral inoculum was added to the cells. After 1 h, the supernatant was aspirated, the wells were washed twice with PBS, and DMEM (Cultilab, Campinas, SP, BR) supplemented with 2% FBS (Gibco—Thermo Fisher Scientific, Waltham, MA, USA) was added to the cells. Luminescence levels were measured at 16 h.p.i. to analyze the virus replication rates, as explained earlier.

### 3.9. Analysis of the Peptides in the CHIKV Entry Steps

The peptides’ action on CHIKV entry into the cells was analyzed as previously described [54,55]. BHK-21 and Huh-7 cells (1 × 10^4^ per well) were seeded in 96-well white culture plates (Greiner Bio-One, Americana, SP, Brazil) for 24 h. Then, the cells were infected with the virus at an MOI of 0.1 and treated with the peptides at the MNTC simultaneously for 1 h at 37 °C. Subsequently, the supernatant was removed, the cells were washed twice with PBS, and DMEM (Cultilab, Campinas, SP, BR) with 2% FBS (Gibco—Thermo Fisher Scientific, Waltham, MA, USA) was added to each well. The plate was incubated at 37 °C, and the antiviral effect against CHIKV was evaluated at 16 h.p.i. The impact of the peptides in the entry step was measured by luminescence levels.

### 3.10. Analysis of the Peptides on the CHIKV Attachment to the Cells

The effect of the peptides on CHIKV attachment to cells was evaluated as previously described [54,55,57]. BHK-21 and Huh-7 cells (1 × 10^4^ per well) were seeded in 96-well white culture plates (Greiner Bio-One, Americana, SP, Brazil). After 24 h, the plate was incubated at 4 °C for 15 min, and the cells were infected with the virus at an MOI of 0.1 and treated with the peptides at the MNTC simultaneously. The plate was incubated at 4 °C for 1 h. At 4 °C, virions interact with receptors on the cell membrane, but the viral particle is not able to enter the cell [54]. Then, the supernatant was aspirated, the wells were washed twice with PBS, and DMEM (Cultilab, Campinas, SP, Brazil) supplemented with 2% FBS (Gibco—Thermo Fisher Scientific, Waltham, MA, USA) was added. The plate was incubated at 37 °C, and the antiviral activity against CHIKV was evaluated at 16 h.p.i. The inhibitory effect of the peptides on CHIKV attachment to host cells was evaluated via the measurement of NLuc activity.

### 3.11. Analysis of the Peptides on CHIKV Internalization

The activity of the peptides on CHIKV internalization in cells was analyzed as previously described [57]. BHK-21 and Huh-7 cells (1 × 10^4^ per well) were seeded in 96-well white culture plates (Greiner Bio-One, Americana, SP, Brazil). After 24 h, the plate was incubated at 4 °C for 15 min, and the cells were infected with the virus at an MOI of 0.1 for 1 h at 4 °C. Then, the supernatant was removed, and the cells were washed twice with PBS and treated with the peptides at the MNTC for 1 h at 37 °C. Subsequently, the supernatant was aspirated, the cells were again washed twice with PBS, and DMEM (Cultilab, Campinas, SP, BR) with 2% FBS (Gibco—Thermo Fisher Scientific, Waltham, MA, USA) was added. At 16 h post-infection, cells were lysed and luciferase activity was measured.

### 3.12. Evaluation of the Peptides on the CHIKV Post-Entry Steps in Cells

The peptides’ action on the post-entry stages of CHIKV in cells was evaluated as previously described [54,55]. BHK-21 and Huh-7 cells (1 × 10^4^ per well) were seeded in 96-well white culture plates (Greiner Bio-One, Americana, SP, Brazil). After 24 h, the cells were infected with the virus at an MOI of 0.1. After 1 h at 37 °C, the supernatant was removed, and the cells were washed twice with PBS and incubated with the peptides at the MNTC for 16 h. After this period, samples were harvested, and virus replication levels were quantified by luminescence using *Renilla* luciferase Assay Reagent (Promega, Madison, WI, USA).

### 3.13. Statistical Analysis

All the described experiments were performed in three independent events, each of which was carried out in three (cytotoxicity) and four (antiviral activity) technical replicates. The CC_50_ and EC_50_ values were determined by nonlinear regression of the dose-response curves (Log [peptide] × response). The statistical significance was determined employing the paired Student’s *t*-test for parametric results and the Mann–Whitney test for nonparametric data using GraphPad Prism 5.0 software (GraphPad Software, San Diego, CA, USA). A *p*-value < 0.05 was considered statistically significant.

## 4. Conclusions

Our study reports the first inhibitory effects of the peptide GA-Hecate and its analogs PSSct1905 and PSSct1910 against CHIKV infection in BHK-21 and Huh-7 cells. Antiviral activities were observed when the peptides were used as prophylactic treatment and/or were administered to CHIKV-infected cells at early and later stages of infection. These peptides exhibit potential for further development into anti-CHIKV drugs, which could help combat this mosquito-borne viral infection.

## Figures and Tables

**Table 1:** General characteristics and biological properties for the cationic peptides GA-Hecate, PSSct1905, and PSSct1910. MW: molecular weight. MNTC: maximum nontoxic concentration. CC_50_: 50% cytotoxic concentration. EC_50_: 50% effective concentration. SI: selectivity index.

					BHK-21 Cells			Huh-7 Cells		
Peptide	Length	MW	Net Charge	MNTC (µM)	CC_50_ (µM)	EC_50_ (µM)	SI	CC_50_ (µM)	EC_50_ (µM)	SI
GA-Hecate	23	2688.43	+9	12.5	23.3	5.7	4.1	221	4.5	49.1
PSSct1905	15	1808.25	+4	12.5	56.5	8.4	6.7	205	7.1	28.9
PSSct1910	23	2684.17	+9	1.6	5.4	1.1	4.9	7.7	0.2	38.5

## Data Availability

Data is contained within the article and supplementary material.

## Supplementary Materials

The following supporting information can be downloaded at: https://www.mdpi.com/article/10.3390/ph16101389/s1, Figure S1. Cytotoxicity of the peptides in BHK-21 and Huh-7 cells. Peptides were incubated at different concentrations (1.6 to 100 µM) in BHK-21 and Huh-7 cells. After 24 h, the cell viability was analyzed using the MTT method. The percentage values are expressed in relation to the results obtained in cells treated with the control (C, sterile water). (A) GA-Hecate in BHK-21 cells. (B) PSSct1905 in BHK-21 cells. (C) PSSct1910 in BHK-21 cells. (D) GA-Hecate in Huh-7 cells. (E) PSSct1905 in Huh-7 cells. (F) PSSct1910 in Huh-7 cells.
