# Supplementary material for: The Synthetic Peptide GA-Hecate and Its Analogs Inhibit Multiple Steps of the Chikungunya Virus Infection Cycle In Vitro
Source: Pharmaceuticals (Basel). 2023 Sep 30;16(10):1389. doi: 10.3390/ph16101389 (PMC10610090; doi:10.3390/ph16101389)
Supplement: Supplementary file 1 [file pharmaceuticals-16-01389-s001.zip › pharmaceuticals-2533501-supplementary.pdf]

*Supplementary Material*

# The Synthetic Peptide GA-Hecate and Its Analogs Inhibit Multiple Steps of the Chikungunya Virus Infection Cycle In Vitro

**Gabriela Miranda Ayusso [1,†], Paulo Ricardo da Silva Sanches [2,*,†], Tamara Carvalho [1], Igor Andrade Santos [3], Daniel Oliveira Silva Martins [1,3], Maria Letícia Duarte Lima [1], Pâmela Jóyce Previdelli da Conceição [1], Cíntia Bittar [1,4], Andres Merits [5], Eduardo Maffud Cilli [6], Ana Carolina Gomes Jardim [1,3], Paula Rahal [1] and Marilia Freitas Calmon [1,*]**

1 Institute of Biosciences, Letters and Exact Sciences, São Paulo State University, São José do Rio Preto 15054-000, SP, Brazil; gabriela.ayusso@unesp.br (G.M.A.); tamara.carvalho@unesp.br (T.C.); daniel.os.martins@unesp.br (D.O.S.M.); maria-leticia.lima@unesp.br (M.L.D.L.); pamela.joyce@unesp.br (P.J.P.d.C.); cintia.bittar@unesp.br (C.B.); jardim@ufu.br (A.C.G.J.); p.rahal@unesp.br (P.R.)

2 School of Pharmaceutical Sciences, São Paulo State University, Araraquara 14800-903, SP, Brazil

3 Institute of Biomedical Sciences, Federal University of Uberlândia, Uberlândia 38408-100, MG, Brazil; igorsantos_12@ufu.br

4 Laboratory of Molecular Immunology, The Rockefeller University, New York, NY 10065, USA

5 Institute of Technology, University of Tartu, 50090 Tartu, Estonia; andres.merits@ut.ee

6 Institute of Chemistry, São Paulo State University, Araraquara 14800-060, SP, Brazil; eduardo.cilli@unesp.br

* Correspondence: paulo.sanches@unesp.br (P.R.d.S.S.); marilia.calmon@unesp.br (M.F.C.)

† These authors contributed equally to this work.

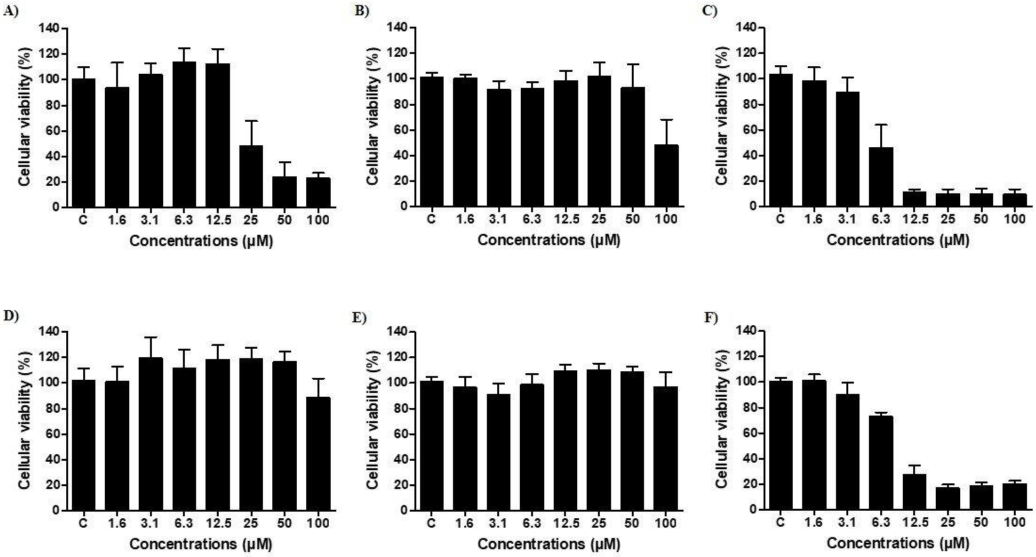


**Figure S1.** Cytotoxicity of the peptides in BHK-21 and Huh-7 cells. Peptides were incubated at different concentrations (1.6 to 100 µM) in BHK-21 and Huh-7 cells. After 24 hours, the cell viability was analyzed using MTT method. The percentage values are expressed in relation to the results obtained in cells treated with the control (**C**, sterile water). **(A)** GA-Hecate in BHK-21 cells. **(B)** PSSct1905 in BHK-21 cells. **(C)** PSSct1910 in BHK-21 cells. **(D)** GA-Hecate in Huh-7 cells. **(E)** PSSct1905 in Huh-7 cells. **(F)** PSSct1910 in Huh-7 cells.
